# Association between Cardiorespiratory Fitness and Circulating Proteins in 50-Year-Old Swedish Men and Women: a Cross-Sectional Study

**DOI:** 10.1186/s40798-021-00343-5

**Published:** 2021-07-26

**Authors:** Malin Enarsson, Tobias Feldreich, Liisa Byberg, Christoph Nowak, Lars Lind, Johan Ärnlöv

**Affiliations:** 1 Center for Clinical Research Dalarna, Uppsala University, Region Dalarna, Nissers väg 3, 79182 Falun, Sweden; 2 School of Health and Social Studies, Dalarna University, 79188 Falun, Sweden; 3 Department of Surgical Sciences, Orthopedics, Uppsala University, Dag Hammarskjölds väg 14, B 75185 Uppsala, Sweden; 4 Division of Family Medicine and Primary Care, Department of Neurobiology, Care Sciences and Society (NVS), Karolinska Institutet, Alfred Nobels Allé 23, 14183 Huddinge, SE Sweden; 5 Department of Medical Sciences, Uppsala University, Dag Hammarskölds väg 10B, 75237 Uppsala, Sweden

**Keywords:** Biomarkers, Proteomics, Cardiorespiratory fitness

## Abstract

**Background and Aims:**

A strong cardiorespiratory fitness is suggested to have beneficial effects on cardiovascular risk; the exact mechanisms underlying the cardioprotective effects of fitness remain uncertain. Our aim was to investigate associations between cardiorespiratory fitness and multiple plasma proteins, in order to obtain insights about physiological pathways associated with the effects of exercise on cardiovascular health.

**Methods:**

In the Prospective investigation of Obesity, Energy and Metabolism (POEM) study (*n*=444 adults aged 50 years, 50% women), cardiorespiratory fitness was measured by a maximal exercise test on bicycle ergometer with gas exchange (VO_2_peak) normalized for body lean mass (dual-energy X-ray absorptiometry (DXA)). We measured 82 cardiovascular proteins associated with cardiovascular pathology and inflammation in plasma samples with a proximity extension assay.

**Results:**

In sex-adjusted linear regression, VO_2_peak was associated with 18 proteins after Bonferroni correction for multiple testing (*p*<0.0006). Following additional adjustment for fat mass (DXA), fasting glucose (mmol/L), low-density lipoprotein (LDL, mmol/L), smoking status, waist/hip ratio, blood pressure (mmHg), education level, and lpnr (lab sequence number), higher VO_2_peak was significantly associated with lower levels of 6 proteins: fatty-acid binding protein-4 (FABP4), interleukin-6 (IL-6), leptin, cystatin-B (CSTB), interleukin-1 receptor antagonist (IL-1RA), and growth differentiation factor 15 (GDF-15), and higher levels of 3 proteins: galanin, kallikrein-6 (KLK6), and heparin-binding EGF-like growth factor (HB-EGF), at nominal *p*-values (*p*<0.05).

**Conclusions:**

We identified multiple novel associations between cardiorespiratory fitness and plasma proteins involved in several atherosclerotic processes and key cellular mechanisms such as inflammation, energy homeostasis, and protease activity, which shed new light on how exercise asserts its beneficial effects on cardiovascular health. Our findings encourage additional studies in order to understand the underlying causal mechanisms for these associations.

**Supplementary Information:**

The online version contains supplementary material available at 10.1186/s40798-021-00343-5.

## Key Points

- The underlying mechanisms for the protective effects of cardiorespiratory fitness on cardiovascular health are incompletely understood.

- We report associations between cardiorespiratory fitness, measured by a maximal exercise test on bicycle ergometer with gas exchange (VO_2_peak), and 82 plasma proteins in a community-based sample with 50-year-old participants.

- Multiple novel associations were found between cardiorespiratory fitness and plasma proteins involved in several aspects of the atherosclerotic process and key cellular mechanisms such as inflammation, energy homeostasis, and protease activity. Results might bring new insights into how exercise asserts its beneficial effects on cardiovascular health.

## Introduction

Cardiorespiratory fitness is a strong and independent predictor of cardiovascular disease (CVD) and all-cause mortality [1–8]. Cardiorespiratory fitness has been shown to be a more powerful predictor of CVD compared to traditional risk factors, e.g., hypertension, dyslipidemia, metabolic syndrome, diabetes, smoking, and leisure time physical activity [5, 9, 10]. It is a diagnostic and prognostic health indicator in the clinical setting and a measure of habitual physical activity [7, 11–13]. Cardiorespiratory fitness is largely modified by physical activity and exercise, but even though these concepts are closely related, they are considered two distinct components of cardiovascular health [7, 9, 14–16]. To a large degree, cardiorespiratory fitness is also determined by genetic factors, and the trainability of cardiorespiratory fitness varies greatly between individuals [17–19].

A strong cardiorespiratory fitness is suggested to have beneficial effects on cardiovascular risk factors such as diabetes [20], hypertension [21, 22], dyslipidemia [23], metabolic syndrome, and obesity [12, 24–27]. These beneficial effects appear independent of weight reduction, as studies have shown reduced risk of CVD and all-cause mortality among individuals regardless of weight loss [10, 16, 26, 28, 29]. Previously, it has been shown that cardiorespiratory fitness largely negates the adverse effects of obesity [4, 15, 30–32]. There are also many health benefits acutely after exercise, which may not be mediated by increased cardiorespiratory fitness [26, 33]. The exact mechanisms underlying cardioprotective effects of fitness remain uncertain [34]. It has been suggested that a high cardiorespiratory fitness may influence levels of circulating proteins involved in the development of CVD. For example, poor cardiorespiratory fitness has been associated with elevated proinflammatory markers, such as C-reactive protein (CRP) and interleukin-6 (IL-6) [35–38] and proteins involved in lipid metabolism and atherosclerotic processes [26, 34, 39].

Novel antibody-based multiplex assays permit quantification of a large number of plasma proteins (proteomics) and have spurred interest in discovering currently unknown disease mechanisms and biomarkers for CVD. However, to date, reports of studies investigating the associations between proteomics and physical activity or cardiorespiratory fitness are scarce. Given the close interplay between proteins involved with inflammation and CVD, the protein panel used (Proseek CVD 1, Olink) could be of particular relevance [26, 34, 39]. This study might shed new light on how exercise asserts its beneficial effects on cardiovascular health. To identify potential relationships between cardiorespiratory fitness and proteins involved in CVD will advance our understanding and potentially lead to advancement in pharmaceutical and interventional treatments in the future. Findings may further encourage additional experimental studies in order to understand the underlying causal mechanisms for these associations.

We hypothesized that higher cardiorespiratory fitness could have associations with levels of plasma proteins involved in cardiovascular disease pathology and inflammation and implicate the development of CVD. In a community-based cohort of Swedish adults, we investigated the association between cardiorespiratory fitness, assessed by a maximal exercise test on bicycle ergometer with gas exchange (VO_2_peak), and plasma levels of 82 cardiovascular proteins measured by a multiplex proteomics assay.

## Materials and Methods

In the Prospective investigation of Obesity, Energy and Metabolism (POEM) study, 502 persons (249 women, 253 men) aged 50 years were assessed in 2009–2015 in Uppsala, Sweden. Inhabitants of Uppsala city were randomly invited to participate 1 month following their 50th birthday; the participation rate was 26%. Participants were included if they were self-reported as healthy and physically able to carry out the bicycle exercise test. Details of the cohort have previously been reported [40]; no exclusion criteria were used. There was no data on ethnicity. The study was approved by the Ethical Board at Uppsala University (Dnr 2009/057) and conducted in accordance with the standards of ethics outlined in the Declaration of Helsinki.

A bicycle exercise test was performed in a random sample of 454 participants in POEM, 444 of whom had complete proteomics and covariate data, and thus comprise the present study sample.

All study participants provided written informed consent. No medication was allowed in the morning. Blood samples of a total of 150ml were obtained in the morning after an overnight fast. Blood was collected in EDTA-plasma tubes that were kept cool during spinning (5min 3000rpm). Analyses of traditional lipids and fasting glucose [41] were conducted immediately, and plasma was stored at −80 °C for later proteomic analyses. Blood pressure was measured in duplicates after 30 min of supine rest. All participants completed a questionnaire about lifestyle, history of diseases, medication, and smoking habits. None of the participants showed any cardiac, pulmonary, or neuromuscular disease that would restrict the work performance.

### Cardiopulmonary Fitness

Using a bicycle ergometer (Jaeger Oxygen Pro, Vyaire Medical, USA), a maximal exercise test with gas exchange (VO_2_peak) was performed in all participants. The same cycle ergometer and metabolic cart were used for every participant. The workload was increased by 10W/min, starting at 30W for women and 50W for men, until exhaustion. The VO_2_peak was recorded and normalized for lean mass (dual-energy X-ray absorptiometry (DXA)) for further calculations.

### Dual-energy X-ray absorptiometry (DXA)

Total and regional body fat and lean mass were estimated using dual-energy X-ray absorptiometry (DXA; Lunar Prodigy, GE Healthcare). In order to minimize the potential operator bias, all scans were performed by the same experienced clinician. By triple measurements in 15 subjects with repositioning according to recommendations from the International Society for Clinical Densitometry, the precision error of the DXA measurements in our laboratory has been calculated. Total fat and lean mass had a precision error of 1.5% and 1.0%, respectively. For analysis, the automatic edge detection was always used; however, all scans were thoroughly checked for errors and manually corrected if needed.

### Proteomics

Proteins with established or presumed role in CVD were measured in plasma by a proximity extension assay (PEA) [42] (Proseek CVD 1, Olink, Uppsala, Sweden). The assay (CVD I) measures 92 proteins and is performed in a 96-well microplate with 92 pairs of oligonucleotide-labeled antibodies and four internal controls. Upon binding their specific target protein, the oligonucleotide functions as a distinctive reporter sequence, which is amplified and quantified with a Fluidigm BiomarkTM HD real-time polymerase chain reaction (PCR) platform. The assay measures relative concentrations (instead of absolute levels) adjusted for plate and negative controls to remove any influence of drift in measurements between plates. Following log2-transformation, protein data were given on a SD-scale for comparison of the results between the proteins [42, 43]. After performing quality control (QC), 10 proteins showed a call rate <75% and were therefore excluded from analyses, leaving 82 proteins for further analysis. Measurements below the level of detection (LOD) were imputed as LOD/2^0.5^. The proteins used in the analyses are shown in Supplementary Figure 1.

### Statistical Analyses

All statistical analyses in the present paper are cross-sectional. VO_2_peak normalized for lean mass by DXA measures was considered the primary exposure (independent variable). All protein data were log-transformed to give a normal distribution. All continuous variables were tested for normality testing by Shapiro-Wilk test. Fasting glucose did not meet normal distribution and was consequently log-transformed to promote normality. In both primary and secondary analyses, separate linear regression models were fitted with the relative concentration of individual proteins as the dependent variable. In all regression analyses, both the exposure and outcome were expressed per standard deviation (SD) increase. In the primary sex-adjusted model, only associations that met the Bonferroni corrected *p*-value threshold (0.05/82= 0.00061) was considered statistically significant. In secondary analyses, we constructed a directed acyclic graph (DAG) [44] to guide the choice of suitable covariates, and investigated the association between the exposure and the proteins (continuous) adjusted for sex (dichotomous), fat mass (DXA, continuous), low-density lipoprotein (LDL, continuous), glucose (continuous), systolic blood pressure (continuous), smoking (dichotomous), waist-hip ratio (WHR, continuous), education level (based on a questionnaire, ordinal), and lab sequence number (ordinal). Only proteins with statistically significant associations in the primary analyses were included in the analyses, and a nominal *p*-value (<0.05) was considered significant. In order to limit the risk of multicollinearity bias in our multivariable modelling, we performed variance inflation factor (VIF) analyses, which measure the correlation and strength of correlation between the explanatory variables in a regression model (Supplementary table 1). Furthermore, we performed tests for effect modification by sex by introducing a multiplicative interaction term in the sex-adjusted analyses. Spearman’s correlation coefficient for the 9 significant proteins was calculated, presented in scatterplots in Supplement Figures 2-10. Statistical software STATA 15 (Stata Corp, College Station, TX) was used in all analyses.

## Results

Baseline characteristics stratified by sex are shown in Table 1. In sex-adjusted linear regression, higher VO_2_peak normalized for lean mass was associated with lower levels of 13 proteins: fatty-acid binding protein-4 (FABP4), interleukin-6 (IL-6), leptin, cystatin-B (CSTB), interleukin-1 receptor antagonist (IL-1RA), growth differentiation factor-15 (GDF-15), tissue-type plasminogen activator (tPA), cathepsin-D (CTSD), hepatocyte growth factor (HGF), e-selectin (SELE), follistatin (FS), fibroblast growth factor-23 (FGF23), tumor necrosis factor ligand superfamily member 14 (TNFSF14); and higher levels of 5 proteins: galanin (GAL), kallikrein-6 (KLK6), heparin-binding EGF-like growth factor (HB-EGF), stem cell factor (SCF), and vascular endothelial growth factor D (VEGF-D), after taking the multiple testing into account using Bonferroni correction (*p*<0.0006, Table 2). The association between VO_2_peak and all 82 proteins are presented in Supplementary Figure 1.

**Table 1:** Baseline characteristics

Variable	Women	Men
Number of participants	220	220
VO2peak (L/min)	1.85±0.4	2.8±0.5
VO2peak (L/min)*	0.05±0.01	0.04±0.01
Fat mass (%)	33.6±7	22.2±5.4
Waist-hip ratio (WHR)	0.87±0.08	0.94±0.05
Body mass index (BMI, kg/m^2^)	26±4.8	26.7±3.5
BMI <18.5 (kg/m^2^)	1 (1%)	0
BMI 18.5–25 (kg/m^2^)	105 (48%)	78 (35%)
BMI 25–30 (kg/m^2^)	78 (35%)	105 (48%)
BMI >30 (kg/m^2^)	36 (16%)	37 (17%)
Systolic blood pressure (mmHg)	120±16	124±15
Diastolic blood pressure (mmHg)	77±10	80±10
Fasting glucose (mmol/L)	5.0±0.5	5.1±1.3
Low density lipoprotein (LDL) (mmol/L)	3.3±0.8	3.6±0.9
Current smoker	25 (11%)	12 (5%)
Education		
<10 years	8 (4%)	21 (10%)
10–12 years	93 (42%)	95 (43%)
>12 years	119 (54%)	104 (47%)

Continuous variables are given as mean ± SD. Dichotomous or ordinal as *n* (proportion %)

*Normalized for lean mass (DXA)

**Table 2:** The associations between VO2peak normalized for lean mass, and the circulating proteins: sex- and multivariable adjusted linear regression. Only proteins that met the Bonferroni adjusted threshold (*p*<0.0006) in the primary sex adjusted analyses are presented. Proteins that met *p*<0.05 threshold in the multivariable-adjusted analyses are highlighted in bold

Protein	Sex-adjusted				Multivariable-adjusted^†^			
	beta	95% CI	95% CI	*p*-value	beta	95% CI	95% CI	*p*-value
		low	high			low	high	
**Fatty acid-binding protein 4 (FABP4)**	−.41	−.49	−.32	<0.0001	−.17	−.25	−.09	<0.0001
**Interleukin-6 (IL-6)**	−.34	−.43	−.25	<0.0001	−.21	−.31	−.10	0.00015
**Leptin**	−.36	−.43	−.29	<0.0001	−.09	−.15	−.04	0.001
**Galanin**	.25	.16	.35	<0.0001	.18	.07	.29	0.002
**Cystatin-B (CSTB)**	−.31	−.40	−.21	<0.0001	−.16	−.27	−.05	0.0046
**Interleukin-1 receptor antagonist protein (IL-1RA)**	−.39	−.48	−.30	<0.0001	−.13	−.23	−.03	0.0085
**Kallikrein-6 (KLK6)**	.18	.08	.27	0.0003	.15	.03	.26	0.012
**Heparin-binding EGF-like growth factor (HB-EGF)**	.18	.08	.28	0.00029	.12	.01	.24	0.037
**Growth/differentiation factor 15 (GDF-15)**	−.19	−.29	−.09	0.00013	−.11	−.23	.00	0.049
Tissue-type plasminogen activator (t-PA)	−.33	−.42	−.24	<0.0001	−.10	−.19	.00	0.054
Cathepsin D (CTSD)	−.26	−.35	−.17	<0.0001	−.04	−.14	.06	0.46
Hepatocyte growth factor (HGF)	−.25	−.35	−.16	<0.0001	−.02	−.13	.08	0.67
E-selectin (SELE)	−.23	−.33	−.14	<0.0001	−.07	−.18	.04	0.20
Stem cell factor (SCF)	.23	.13	.32	<0.0001	.10	−.01	.22	0.073
Vascular endothelial growth factor D (VEGF-D)	.18	.09	.27	<0.0001	.06	−.04	.16	0.26
Follistatin (FS)	−.19	−.29	−.09	0.00012	−.07	−.18	.05	0.25
Fibroblast growth factor 23 (FGF-23)	−.17	−.27	−.08	0.0004	−.10	−.21	.01	0.084
Tumor necrosis factor ligand superfamily member 14 (TNFSF14)	−.17	−.27	−.07	0.00057	.04	−.07	.15	0.47

^†^Multivariable linear regression further adjusted for fat mass (DXA), fasting glucose (mmol/L), LDL (mmol/L), WHR, smoking status, blood pressure (mmHg), educational level, and lpnr (lab test sequence number)

After further adjusting for fat mass (DXA), fasting glucose (mmol/L), low-density lipoprotein (LDL, mmol/L), smoking status, waist/hip ratio (WHR), blood pressure (mmHg), education level, and lpnr (lab sequence number), VO_2_peak normalized for lean mass was still significantly associated with 9 of the above proteins: FABP4, IL-6, leptin, galanin, CSTB, IL-1RA, KLK6, HB-EGF, and GDF-15 (Table 2). Spearman’s correlation coefficients for the 9 significant proteins range between −0.39 and 0.26 (Supplementary Figures 2-10). When we included all 9 significant proteins in the same multivariable model, the R2 value for the whole model was 0.27. When we added clinical covariates to the above model (sex, fat mass, LDL, glucose, systolic blood pressure, smoking, WHR, and education level), the R2 value for the whole model was 0.45. In the multivariable modeling, variance inflation factor (VIF), no evidence of multicollinearity was found. Multiplicative interaction analyses suggest that there was an effect modification by sex for leptin, FGF23, CTSD, and FABP4, results presented in Supplementary Table 2.

## Discussion

### Principal Findings

The present community-based study showed that higher cardiorespiratory fitness, assessed by VO_2_peak, was significantly associated with lower plasma levels of 6 proteins (FABP4, IL-6, leptin, CSTB, IL-1RA, GDF-15), and higher levels of 3 proteins (galanin, KLK6, HB-EGF) independent of cardiovascular risk factors and fat mass. Interestingly, the combination of these 9 explained 27% of the variance of cardiorespiratory fitness.

### Comparison with the Literature

Although there is an increasing interest in the use of proteomics in the CVD field, we are not aware of any previous study reporting associations between cardiorespiratory fitness and multiplex proteomics of blood plasma in the general population. However, we are aware of one previous community-based study that investigated associations between proteomics of blood plasma and physical activity measured by self-reported leisure time by Stattin et al. [39]. In that study, leisure time physical activity was consistently associated with four cardiovascular proteins independent of body fat in two community-based cohorts with more than 6000 participants. Of these proteins, three were also associated with cardiorespiratory fitness in our study: CSTB, FAPB4, IL-1RA. Cardiorespiratory fitness has been suggested as a more reliable marker of habitual physical activity compared to questionnaire-based data [7, 45], but other studies show that cardiorespiratory fitness and physical activity may represent some distinct and independent associations with cardiovascular health [7]. Whether the differences between the present study and the study by Stattin et al. is due to specific differences in the effects on cardiovascular health between cardiorespiratory fitness and physical activity, or whether this discrepancy is due to differences in study populations, proteomic panels and study design between the two studies remains to be established. As the replication cohort in Stattin et al. consisted of only women, our study, that included both men and women, represents the first replication of some of their findings in men.

There is also one previous study that has investigated cardiorespiratory fitness and protein expression in skeletal muscle in trained and untrained individuals [46]. They identified 92 proteins that differed in a muscle of trained compared to untrained at rest, and also found differences in long-term and acute changes of proteins involved in energy metabolism.

### Potential Mechanisms

We used cross-sectional observational data, and no firm conclusions regarding underlying mechanisms or causality can be drawn from our results. Results are modest at best, and cardiorespiratory fitness accounted for a limited degree of the variance measured. Yet, there are several possible explanations for the association between cardiorespiratory fitness and the specific proteins in our study:

First, the inverse association between better cardiorespiratory fitness and lower levels of the inflammatory proteins IL-6 and IL-1RA support the notion of an anti-inflammatory effect of cardiorespiratory fitness. Even though exercise is linked to an acute transient inflammatory response, an abundance of studies show that exercise may exert long-term cardioprotective effects by lowering chronic inflammation [34, 47–52]. There is increasing recognition of inflammation as a key pathogenic mechanism in atherosclerosis and the development of cardiovascular disease [50, 53–57]. Adipose tissue has been suggested to play a key pro-inflammatory role in mediating associations between physical activity and inflammation [47, 58]. In the present study, cardiorespiratory fitness was associated with both IL-6 and IL-1RA independent of body fat, indicating other potential pathways as previously discussed [59].

Second, we found association between cardiorespiratory fitness and three proteins involved in energy homeostasis; specifically, we found inverse relationships with the adipokines leptin and FABP-4 and positive associations with the peptide hormone galanin. These associations remained significant after adjustment for body fat mass and WHR. *FABP-4* and leptin are hormonal bioactive molecules secreted by adipose tissue that control energy balance, have been linked to metabolic and inflammatory pathways, and are associated with the development of insulin resistance and atherosclerosis [60–62]. Higher levels of physical activity and cardiorespiratory fitness are associated with decreased FABP-4 and leptin levels largely driven by a decrease in body fat, although associations appear to be independent of weight loss [39, 63–67]. The gut and central nervous system-derived hormone galanin exerts metabolic effects including regulating glucose metabolism and involved metabolic syndrome by alleviating insulin resistance [68]. In experimental studies, exercise upregulates galanin expression, resulting in improvement in insulin sensitivity [69]. All three proteins might modulate CVD risk by being involved in insulin resistance, and higher levels of FABP-4 and leptin have been associated with cardiac pathophysiology and poorer cardiovascular outcome [61, 70, 71].

Third, the positive association between cardiorespiratory fitness and the protease KLK6 and the inverse association between VO_2_peak and the protease inhibitor CSTB indicate a role for cardiorespiratory fitness in the regulation of protease activity. Protease activity and its inhibition appear to play key roles in the atherosclerotic process [72–74]. Higher levels of physical activity in adults and higher fitness in children have been associated with lower CSTB levels [39, 75, 76]. KLK6 and CSTB have been suggested as biomarkers reflecting cardiovascular disease [39, 75–77] and neurodegenerative diseases [78–80].

Fourth, higher cardiorespiratory fitness was associated with higher levels of HB-EGF. HB-EGF is an epidermal growth factor that is one of the ligands that activates epidermal growth factor receptor (EGFR), which regulates key cellular processes of cell biology, including migration and proliferation of vascular smooth muscle, tissue homeostasis, and tumorigenesis [81–83]. EGFR signaling by its ligands is essential for normal cardiac development and may have the potential to modulate the function of cells involved in the atherosclerotic process [82, 84]. Increased plasma HB-EGF has been associated with obesity and coronary artery disease [85, 86]; however, no studies have addressed whether modulation of EGFR signaling impacts atherogenesis [82]. HB-EGF function and its role in cardiovascular biology and disease pathogenesis are only beginning to be elucidated. One study has previously shown that HB-EGF is upregulated in skeletal muscle by exercise and has been proposed to contribute glucose homeostasis and insulin sensitivity [87]. Findings highlight the complexity of cardiovascular biology, and more research is needed to understand this relationship.

Fifth, cardiorespiratory fitness had a negative association with GDF-15. GDF-15 is a member of the transforming growth factor-β cytokine superfamily. It is believed to have a role in energy balance and glucose homeostasis. Increased levels are associated with cardiovascular disease such as atherosclerosis, obesity, insulin resistance, and diabetes [88–91]. It is a strong predictor of all-cause mortality [92, 93]. GDF-15 has been suggested to be a marker for those at risk for diabetes or obesity and is proposed as an emerging cardiometabolic biomarker [91, 94, 95]. To our knowledge there are no studies that have investigated cardiorespiratory fitness association with GDF-15. In small studies, higher levels of GDF-15 have been observed directly after exercise and exercise interventions, however, it is currently unclear how exercise-induced alternations in GDF 15 might be associated with metabolic improvements following exercise training [96, 97]. In this study higher level of cardiorespiratory fitness is associated with lower levels of GDF-15, adding novel knowledge to the field.

Finally, analyses suggest that there may be some effect modification by sex between leptin, FGF23, CSTD, and FABP4 and fitness, and additional studies are warranted to confirm whether the underlying biology for these associations are different in men and women.

### Strength and Limitations

Strengths of the study include the high-quality phenotyping of study participants and the use of objective methods to assess cardiorespiratory fitness and body composition. A state-of-the art antibody-based proteomics assays with rigorous quality control steps was used to measure proteins. Limitations include the use of a multiplex proteomic assay limited to 82 plasma proteins that were primarily designed to be relevant for cardiovascular disease pathology and inflammation. Additional studies, using larger untargeted proteomic platforms are warranted to get additional insights on the interplay between cardiorespiratory fitness and the circulating proteome. The moderately sized study sample consisting of 50-year-old healthy men and women living in the region of Uppsala limits the generalizability of findings. Due to the cross-sectional design, it is not possible to test causal effects of the proteins, and the observed associations could be due to causation, confounding, or reverse causation. Tissue of origin and regulation of the circulating levels of the proteins in the present study is largely unknown. The proteomics assays provided relative, rather than absolute concentrations, which make direct comparisons with other studies difficult. Another limitation is that this study cannot differentiate between genetically determined cardiorespiratory fitness from fitness due to an increased level of physical activity.

## Conclusion

We identified multiple novel associations between cardiorespiratory fitness (VO_2_peak) and plasma proteins involved in several aspects of the atherosclerotic process and key cellular mechanisms such as inflammation, energy homeostasis, and protease activity, which may bring new insights into how exercise asserts its beneficial effects on cardiovascular health. We are not aware of any previous study reporting association between cardiorespiratory fitness and multiplex proteomics of blood plasma in the general population. Our findings will hopefully advance our knowledge and lead to further studies. Our findings encourage additional studies in order to understand the underlying causal mechanisms for these associations.

## Supplementary Information

**Additional file 1.** Supplementary figure 1.

**Additional file 2.** Supplementary figures 2–10.

**Additional file 3.** Supplementary table 1.

**Additional file 4.** Supplementary table 2.

## Data Availability

Data can be made available to researcher upon application to Principal Investigator Lars Lind (lars.lind@medsci.uu.se).

## References

[1] Blair SN, Kampert JB, Kohl HW, Barlow CE, Macera CA, Paffenbarger RS (1996). Influences of cardiorespiratory fitness and other precursors on cardiovascular disease and all-cause mortality in men and women. Jama..

[2] Kodama S, Saito K, Tanaka S, Maki M, Yachi Y, Asumi M, Sugawara A, Totsuka K, Shimano H, Ohashi Y, Yamada N, Sone H (2009). Cardiorespiratory fitness as a quantitative predictor of all-cause mortality and cardiovascular events in healthy men and women: a meta-analysis. JAMA..

[3] Laukkanen JA, Kunutsor SK, Yates T, Willeit P, Kujala UM, Khan H, Zaccardi F (2020). Prognostic relevance of cardiorespiratory fitness as assessed by submaximal exercise testing for all-cause mortality: a UK Biobank prospective study. Mayo Clin Proc.

[4] Sui X, Sarzynski MA, Lee DC, Kokkinos PF (2017). Impact of changes in cardiorespiratory fitness on hypertension, dyslipidemia and survival: an overview of the epidemiological evidence. Prog Cardiovasc Dis.

[5] Myers J, Prakash M, Froelicher V, Do D, Partington S, Atwood JE (2002). Exercise capacity and mortality among men referred for exercise testing. N Engl J Med.

[6] Myers J, Kaykha A, George S, Abella J, Zaheer N, Lear S, Yamazaki T, Froelicher V (2004). Fitness versus physical activity patterns in predicting mortality in men. Am J Med.

[7] DeFina LF, Haskell WL, Willis BL, Barlow CE, Finley CE, Levine BD (2015). Physical activity versus cardiorespiratory fitness: two (partly) distinct components of cardiovascular health?. Prog Cardiovasc Dis.

[8] Williams PT (2001). Physical fitness and activity as separate heart disease risk factors: a meta-analysis. Med Sci Sports Exerc.

[9] Ozemek C, Laddu DR, Lavie CJ, Claeys H, Kaminsky LA, Ross R, Wisloff U, Arena R, Blair SN (2018). An update on the role of cardiorespiratory fitness, structured exercise and lifestyle physical activity in preventing cardiovascular disease and health risk. Prog Cardiovasc Dis.

[10] Lee DC, Sui X, Ortega FB, Kim YS, Church TS, Winett RA, Ekelund U, Katzmarzyk PT, Blair SN (2011). Comparisons of leisure-time physical activity and cardiorespiratory fitness as predictors of all-cause mortality in men and women. Br J Sports Med.

[11] Ross R, Blair SN, Arena R, Church TS, Després JP, Franklin BA, Haskell WL, Kaminsky LA, Levine BD, Lavie CJ, Myers J, Niebauer J, Sallis R, Sawada SS, Sui X, Wisløff U, American Heart Association Physical Activity Committee of the Council on Lifestyle and Cardiometabolic Health, Council on Clinical Cardiology, Council on Epidemiology and Prevention, Council on Cardiovascular and Stroke Nursing, Council on Functional Genomics and Translational Biology, Stroke Council (2016). Importance of assessing cardiorespiratory fitness in clinical practice: a case for fitness as a clinical vital sign: a scientific statement from the American Heart Association. Circulation..

[12] Kaminsky LA, Arena R, Ellingsen Ø, Harber MP, Myers J, Ozemek C, Ross R (2019). Cardiorespiratory fitness and cardiovascular disease - the past, present, and future. Prog Cardiovasc Dis.

[13] Lee DC, Artero EG, Sui X, Blair SN (2010). Mortality trends in the general population: the importance of cardiorespiratory fitness. J Psychopharmacol.

[14] Carbone S, Del Buono MG, Ozemek C, Lavie CJ (2019). Obesity, risk of diabetes and role of physical activity, exercise training and cardiorespiratory fitness. Prog Cardiovasc Dis.

[15] Ortega FB, Cadenas-Sanchez C, Lee DC, Ruiz JR, Blair SN, Sui X (2018). Fitness and fatness as health markers through the lifespan: an overview of current knowledge. Prog Prev Med.

[16] Myers J, McAuley P, Lavie CJ, Despres JP, Arena R, Kokkinos P (2015). Physical activity and cardiorespiratory fitness as major markers of cardiovascular risk: their independent and interwoven importance to health status. Prog Cardiovasc Dis.

[17] Williams CJ, Williams MG, Eynon N, Ashton KJ, Little JP, Wisloff U, Coombes JS (2017). Genes to predict VO(2max) trainability: a systematic review. BMC Genomics.

[18] Bye A, Klevjer M, Ryeng E, Silva G, Moreira JBN, Stensvold D (2020). Identification of novel genetic variants associated with cardiorespiratory fitness. Prog Cardiovasc Dis.

[19] Skinner JS, Wilmore KM, Krasnoff JB, Jaskólski A, Jaskólska A, Gagnon J, Province MA, Leon AS, Rao DC, Wilmore JH, Bouchard C (2000). Adaptation to a standardized training program and changes in fitness in a large, heterogeneous population: the HERITAGE Family Study. Med Sci Sports Exerc.

[20] Regensteiner JG, Sippel J, McFarling ET, Wolfel EE, Hiatt WR (1995). Effects of non-insulin-dependent diabetes on oxygen consumption during treadmill exercise. Med Sci Sports Exerc.

[21] Missault L, Duprez D, de Buyzere M, de Backer G, Clement D (1992). Decreased exercise capacity in mild essential hypertension: non-invasive indicators of limiting factors. J Hum Hypertens.

[22] Kokkinos P, Pittaras A, Narayan P, Faselis C, Singh S, Manolis A (2007). Exercise capacity and blood pressure associations with left ventricular mass in prehypertensive individuals. Hypertension..

[23] Parto P, Lavie CJ, Swift D, Sui X (2015). The role of cardiorespiratory fitness on plasma lipid levels. Expert Rev Cardiovasc Ther.

[24] Berntzen B, Jukarainen S, Kataja M, Hakkarainen A, Lundbom J, Lundbom N, et al. Physical activity, cardiorespiratory fitness, and metabolic outcomes in monozygotic twin pairs discordant for body mass index. Scand J Med Sci Sports. 2017. 10.1111/sms.12975 28833625

[25] Mondal H, Mishra SP (2017). Effect of BMI, body fat percentage and fat free mass on maximal oxygen consumption in healthy young adults. J Clin Diagn Res.

[26] Kokkinos P, Myers J (2010). Exercise and physical activity: clinical outcomes and applications. Circulation..

[27] Carnethon MR, Gidding SS, Nehgme R, Sidney S, Jacobs DR, Liu K (2003). Cardiorespiratory fitness in young adulthood and the development of cardiovascular disease risk factors. Jama..

[28] Lee DC, Sui X, Artero EG, Lee IM, Church TS, McAuley PA (2011). Long-term effects of changes in cardiorespiratory fitness and body mass index on all-cause and cardiovascular disease mortality in men: the Aerobics Center Longitudinal Study. Circulation..

[29] De Schutter A, Lavie CJ, Milani RV (2014). The impact of obesity on risk factors and prevalence and prognosis of coronary heart disease-the obesity paradox. Prog Cardiovasc Dis.

[30] Kokkinos P, Faselis C, Franklin B, Lavie CJ, Sidossis L, Moore H, Karasik P, Myers J (2019). Cardiorespiratory fitness, body mass index and heart failure incidence. Eur J Heart Fail.

[31] Lavie CJ, Schutter AD, Archer E, McAuley PA, Blair SN (2014). Obesity and prognosis in chronic diseases--impact of cardiorespiratory fitness in the obesity paradox. Curr Sports Med Rep.

[32] McAuley PA, Beavers KM (2014). Contribution of cardiorespiratory fitness to the obesity paradox. Prog Cardiovasc Dis.

[33] Kramer A (2020). An overview of the beneficial effects of exercise on health and performance. Adv Exp Med Biol.

[34] Lin X, Zhang X, Guo J, Roberts CK, McKenzie S, Wu WC, et al. Effects of exercise training on cardiorespiratory fitness and biomarkers of cardiometabolic health: a systematic review and meta-analysis of randomized controlled trials. J Am Heart Assoc. 2015;4(7). 10.1161/JAHA.115.002014 PMC4608087 26116691

[35] Mikkelsen UR, Couppe C, Karlsen A, Grosset JF, Schjerling P, Mackey AL (2013). Life-long endurance exercise in humans: circulating levels of inflammatory markers and leg muscle size. Mech Ageing Dev.

[36] Church TS, Barlow CE, Earnest CP, Kampert JB, Priest EL, Blair SN (2002). Associations between cardiorespiratory fitness and C-reactive protein in men. Arterioscler Thromb Vasc Biol.

[37] Kim DJ, Noh JH, Lee BW, Choi YH, Jung JH, Min YK, Lee MS, Lee MK, Kim KW (2005). A white blood cell count in the normal concentration range is independently related to cardiorespiratory fitness in apparently healthy Korean men. Metabolism..

[38] LaMonte MJ, Durstine JL, Yanowitz FG, Lim T, DuBose KD, Davis P (2002). Cardiorespiratory fitness and C-reactive protein among a tri-ethnic sample of women. Circulation..

[39] Stattin K, Lind L, Elmstahl S, Wolk A, Lemming EW, Melhus H (2019). Physical activity is associated with a large number of cardiovascular-specific proteins: cross-sectional analyses in two independent cohorts. Eur J Prev Cardiol.

[40] Lind L, Strand R, Michaëlsson K, Ahlström H, Kullberg J (2020). Voxel-wise study of cohort associations in whole-body MRI: application in metabolic syndrome and its components. Radiology..

[41] Carlsson L, Lind L, Larsson A (2010). Reference values for 27 clinical chemistry tests in 70-year-old males and females. Gerontology..

[42] Assarsson E, Lundberg M, Holmquist G, Bjorkesten J, Thorsen SB, Ekman D (2014). Homogenous 96-plex PEA immunoassay exhibiting high sensitivity, specificity, and excellent scalability. PLoS One.

[43] Lundberg M, Eriksson A, Tran B, Assarsson E, Fredriksson S (2011). Homogeneous antibody-based proximity extension assays provide sensitive and specific detection of low-abundant proteins in human blood. Nucleic Acids Res.

[44] Textor J, van der Zander B, Gilthorpe MS, Liskiewicz M, Ellison GT (2016). Robust causal inference using directed acyclic graphs: the R package ‘dagitty’. Int J Epidemiol.

[45] Sallis JF, Saelens BE (2000). Assessment of physical activity by self-report: status, limitations, and future directions. Res Q Exerc Sport.

[46] Schild M, Ruhs A, Beiter T, Zügel M, Hudemann J, Reimer A, Krumholz-Wagner I, Wagner C, Keller J, Eder K, Krüger K, Krüger M, Braun T, Nieß A, Steinacker J, Mooren FC (2015). Basal and exercise induced label-free quantitative protein profiling of m. vastus lateralis in trained and untrained individuals. J Proteome.

[47] Madssen E, Skaug EA, Wisløff U, Ellingsen Ø, Videm V (2019). Inflammation is strongly associated with cardiorespiratory fitness, sex, BMI, and the metabolic syndrome in a self-reported healthy population: HUNT3 fitness study. Mayo Clin Proc.

[48] Petersen AM, Pedersen BK (2005). The anti-inflammatory effect of exercise. J Appl Physiol (1985).

[49] Beavers KM, Brinkley TE, Nicklas BJ (2010). Effect of exercise training on chronic inflammation. Clin Chim Acta.

[50] Vasan RS (2006). Biomarkers of cardiovascular disease: molecular basis and practical considerations. Circulation..

[51] Swardfager W, Herrmann N, Cornish S, Mazereeuw G, Marzolini S, Sham L (2012). Exercise intervention and inflammatory markers in coronary artery disease: a meta-analysis. Am Heart J.

[52] Lavie CJ, Church TS, Milani RV, Earnest CP (2011). Impact of physical activity, cardiorespiratory fitness, and exercise training on markers of inflammation. J Cardiopulm Rehabil Prev.

[53] Ross R (1999). Atherosclerosis--an inflammatory disease. N Engl J Med.

[54] Ridker PM, Rifai N, Rose L, Buring JE, Cook NR (2002). Comparison of C-reactive protein and low-density lipoprotein cholesterol levels in the prediction of first cardiovascular events. N Engl J Med.

[55] Kaptoge S, Di Angelantonio E, Pennells L, Wood AM, White IR, Gao P (2012). C-reactive protein, fibrinogen, and cardiovascular disease prediction. N Engl J Med.

[56] Holm Nielsen S, Jonasson L, Kalogeropoulos K, Karsdal MA, Reese-Petersen AL, Auf dem Keller U (2020). Exploring the role of extracellular matrix proteins to develop biomarkers of plaque vulnerability and outcome. J Intern Med.

[57] Pearson TA, Mensah GA, Alexander RW, Anderson JL, Cannon RO, Criqui M (2003). Markers of inflammation and cardiovascular disease: application to clinical and public health practice: a statement for healthcare professionals from the Centers for Disease Control and Prevention and the American Heart Association. Circulation..

[58] Lakoski SG, Barlow CE, Farrell SW, Berry JD, Morrow JR, Haskell WL (2011). Impact of body mass index, physical activity, and other clinical factors on cardiorespiratory fitness (from the Cooper Center longitudinal study). Am J Cardiol.

[59] Wedell-Neergaard AS, Krogh-Madsen R, Petersen GL, Hansen ÅM, Pedersen BK, Lund R, Bruunsgaard H (2018). Cardiorespiratory fitness and the metabolic syndrome: roles of inflammation and abdominal obesity. PLoS One.

[60] Furuhashi M (2019). Fatty acid-binding protein 4 in cardiovascular and metabolic diseases. J Atheroscler Thromb.

[61] Rodríguez-Calvo R, Girona J, Alegret JM, Bosquet A, Ibarretxe D, Masana L (2017). Role of the fatty acid-binding protein 4 in heart failure and cardiovascular disease. J Endocrinol.

[62] Martin SS, Qasim A, Reilly MP (2008). Leptin resistance: a possible interface of inflammation and metabolism in obesity-related cardiovascular disease. J Am Coll Cardiol.

[63] Fedewa MV, Hathaway ED, Ward-Ritacco CL, Williams TD, Dobbs WC (2018). The effect of chronic exercise training on leptin: a systematic review and meta-analysis of randomized controlled trials. Sports Med.

[64] Spartano NL, Stevenson MD, Xanthakis V, Larson MG, Andersson C, Murabito JM, Vasan RS (2017). Associations of objective physical activity with insulin sensitivity and circulating adipokine profile: the Framingham Heart Study. Clin Obes.

[65] Lázaro I, Ferré R, Plana N, Aragonès G, Girona J, Merino J, Heras M, Cabré A, Masana L (2012). Lifestyle changes lower FABP4 plasma concentration in patients with cardiovascular risk. Rev Esp Cardiol (Engl Ed).

[66] Maïmoun L, Simar D, Caillaud C, Coste O, Puech AM, Jaussent A, Mariano-Goulart D, Sultan C (2011). Basal plasma leptin levels in healthy elderly males are related to physical fitness without impact on bone metabolism. J Sports Med Phys Fitness.

[67] Sanches RB, Poli VFS, Fidalgo JPN, Andrade-Silva SG, Cerrone LA, Oyama LM, Dâmaso AR, dos Santos RT, Caranti DA (2018). The hyperleptinemia state can downregulate cardiorespiratory fitness and energy expenditure in obese women. Physiol Behav.

[68] Fang P, Yu M, Shi M, Bo P, Zhang Z (2020). Galanin peptide family regulation of glucose metabolism. Front Neuroendocrinol.

[69] Fang P, He B, Shi M, Zhu Y, Bo P, Zhang Z (2015). Crosstalk between exercise and galanin system alleviates insulin resistance. Neurosci Biobehav Rev.

[70] Egbuche O, Biggs ML, Ix JH, Kizer JR, Lyles MF, Siscovick DS (2020). Fatty acid binding protein-4 and risk of cardiovascular disease: the Cardiovascular Health Study. J Am Heart Assoc.

[71] Söderberg S, Ahrén B, Jansson JH, Johnson O, Hallmans G, Asplund K (1999). Leptin is associated with increased risk of myocardial infarction. J Intern Med.

[72] Jormsjö S, Wuttge DM, Sirsjö A, Whatling C, Hamsten A, Stemme S, Eriksson P (2002). Differential expression of cysteine and aspartic proteases during progression of atherosclerosis in apolipoprotein E-deficient mice. Am J Pathol.

[73] Cicha I, Wörner A, Urschel K, Beronov K, Goppelt-Struebe M, Verhoeven E, Daniel WG, Garlichs CD (2011). Carotid plaque vulnerability: a positive feedback between hemodynamic and biochemical mechanisms. Stroke..

[74] Lindstedt KA, Leskinen MJ, Kovanen PT (2004). Proteolysis of the pericellular matrix: a novel element determining cell survival and death in the pathogenesis of plaque erosion and rupture. Arterioscler Thromb Vasc Biol.

[75] Gonçalves I, Hultman K, Dunér P, Edsfeldt A, Hedblad B, Fredrikson GN, Björkbacka H, Nilsson J, Bengtsson E (2016). High levels of cathepsin D and cystatin B are associated with increased risk of coronary events. Open Heart.

[76] Dencker M, Tanha T, Karlsson MK, Wollmer P, Andersen LB, Thorsson O (2017). Cystatin B, cathepsin L and D related to surrogate markers for cardiovascular disease in children. PLoS One.

[77] Darmanis S, Nong RY, Vänelid J, Siegbahn A, Ericsson O, Fredriksson S, Bäcklin C, Gut M, Heath S, Gut IG, Wallentin L, Gustafsson MG, Kamali-Moghaddam M, Landegren U (2011). ProteinSeq: high-performance proteomic analyses by proximity ligation and next generation sequencing. PLoS One.

[78] Stefanini AC, da Cunha BR, Henrique T, Tajara EH (2015). Involvement of Kallikrein-related peptidases in normal and pathologic processes. Dis Markers.

[79] Bayani J, Diamandis EP (2011). The physiology and pathobiology of human kallikrein-related peptidase 6 (KLK6). Clin Chem Lab Med.

[80] Kopitar-Jerala N (2015). The role of Stefin B in neuro-inflammation. Front Cell Neurosci.

[81] Schneider MR, Wolf E (2009). The epidermal growth factor receptor ligands at a glance. J Cell Physiol.

[82] Makki N, Thiel KW, Miller FJ (2013). The epidermal growth factor receptor and its ligands in cardiovascular disease. Int J Mol Sci.

[83] Holbro T, Hynes NE (2004). ErbB receptors: directing key signaling networks throughout life. Annu Rev Pharmacol Toxicol.

[84] Dreux AC, Lamb DJ, Modjtahedi H, Ferns GA (2006). The epidermal growth factor receptors and their family of ligands: their putative role in atherogenesis. Atherosclerosis..

[85] Matsumoto S, Kishida K, Shimomura I, Maeda N, Nagaretani H, Matsuda M, Nishizawa H, Kihara S, Funahashi T, Matsuzawa Y, Yamada A, Yamashita S, Tamura S, Kawata S (2002). Increased plasma HB-EGF associated with obesity and coronary artery disease. Biochem Biophys Res Commun.

[86] Kim S, Subramanian V, Abdel-Latif A, Lee S (2020). Role of heparin-binding epidermal growth factor-like growth factor in oxidative stress-associated metabolic diseases. Metab Syndr Relat Disord.

[87] Fukatsu Y, Noguchi T, Hosooka T, Ogura T, Kotani K, Abe T, Shibakusa T, Inoue K, Sakai M, Tobimatsu K, Inagaki K, Yoshioka T, Matsuo M, Nakae J, Matsuki Y, Hiramatsu R, Kaku K, Okamura H, Fushiki T, Kasuga M (2009). Muscle-specific overexpression of heparin-binding epidermal growth factor-like growth factor increases peripheral glucose disposal and insulin sensitivity. Endocrinology..

[88] Dostálová I, Roubícek T, Bártlová M, Mráz M, Lacinová Z, Haluzíková D (2009). Increased serum concentrations of macrophage inhibitory cytokine-1 in patients with obesity and type 2 diabetes mellitus: the influence of very low calorie diet. Eur J Endocrinol.

[89] Vila G, Riedl M, Anderwald C, Resl M, Handisurya A, Clodi M, Prager G, Ludvik B, Krebs M, Luger A (2011). The relationship between insulin resistance and the cardiovascular biomarker growth differentiation factor-15 in obese patients. Clin Chem.

[90] Eddy AC, Trask AJ (2021). Growth differentiation factor-15 and its role in diabetes and cardiovascular disease. Cytokine Growth Factor Rev.

[91] Adela R, Banerjee SK (2015). GDF-15 as a target and biomarker for diabetes and cardiovascular diseases: a translational prospective. J Diabetes Res.

[92] Daniels LB, Clopton P, Laughlin GA, Maisel AS, Barrett-Connor E (2011). Growth-differentiation factor-15 is a robust, independent predictor of 11-year mortality risk in community-dwelling older adults: the Rancho Bernardo Study. Circulation..

[93] Wiklund FE, Bennet AM, Magnusson PK, Eriksson UK, Lindmark F, Wu L (2010). Macrophage inhibitory cytokine-1 (MIC-1/GDF15): a new marker of all-cause mortality. Aging kCell.

[94] Xu J, Kimball TR, Lorenz JN, Brown DA, Bauskin AR, Klevitsky R, Hewett TE, Breit SN, Molkentin JD (2006). GDF15/MIC-1 functions as a protective and antihypertrophic factor released from the myocardium in association with SMAD protein activation. Circ Res.

[95] Echouffo-Tcheugui JB, Daya N, Matsushita K, Wang D, Ndumele CE, Al Rifai M (2021). Growth differentiation factor (GDF)-15 and cardiometabolic outcomes among older adults: the Atherosclerosis Risk in Communities Study. Clin Chem.

[96] Zhang H, Fealy CE, Kirwan JP (2019). Exercise training promotes a GDF15-associated reduction in fat mass in older adults with obesity. Am J Physiol Endocrinol Metab.

[97] Cai L, Li C, Wang Y, Mo Y, Yin J, Ma X (2021). Increased serum GDF15 related to improvement in metabolism by lifestyle intervention among young overweight and obese adults. Diabetes Metab Syndr Obes.

