# Supplementary material for: Association between Cardiorespiratory Fitness and Circulating Proteins in 50-Year-Old Swedish Men and Women: a Cross-Sectional Study
Source: Sports Med Open. 2021 Jul 26;7:52. doi: 10.1186/s40798-021-00343-5 (PMC8313632; doi:10.1186/s40798-021-00343-5)

**Sports Medicine Open**

**Association between cardiorespiratory fitness and circulating proteins in 50-year-old Swedish men and women.**

**Malin Enarsson**_a_**, Tobias Feldreich**_b_**, Liisa Byberg**_c_**, Christoph Nowak**_d_**, Lars Lind**_e_**, Johan Ärnlöv**_bd_

*_a_ Center for Clinical Research Dalarna, Uppsala University, Region Dalarna, Nissers väg 3, 79182, Falun, Sweden. malinanna.enarsson@regiondalarna.se*

***_b_*** *School of Health and Social Studies, Dalarna University, 79188, Falun Sweden*

*_c_ Department of Surgical Sciences, Orthopeadics, Uppsala University_,_ Dag Hammarskjölds väg 14 B 75185, Uppsala, Sweden.*

*_d_ Division of Family Medicine and Primary Care, Department of Neurobiology, Care Sciences and Society (NVS), Karolinska Institutet, Alfred Nobels Allé 23, SE 14183, Huddinge, Sweden. johan.arnlov@ki.se*

*_e_ Department of Medical Sciences, Uppsala University, Dag Hammarskölds väg 10B 75237, Uppsala, Sweden.*

**Corresponding author**

Johan Ärnlöv

Division of Family Medicine and Primary Care, Department of Neurobiology, Care Sciences and Society (NVS), Karolinska Institutet, Alfred Nobels Allé 23, SE 14183, Huddinge, Sweden.

Email: johan.arnlov@ki.se

**Supplementary Figure 1.** The association between cardiorespiratory fitness, assessed by VO_2_peak normalized for lean mass and 82 plasma proteins. Data are regression coefficients expressed per SD increase for both dependent and independent variables and 95 % confidence intervals.
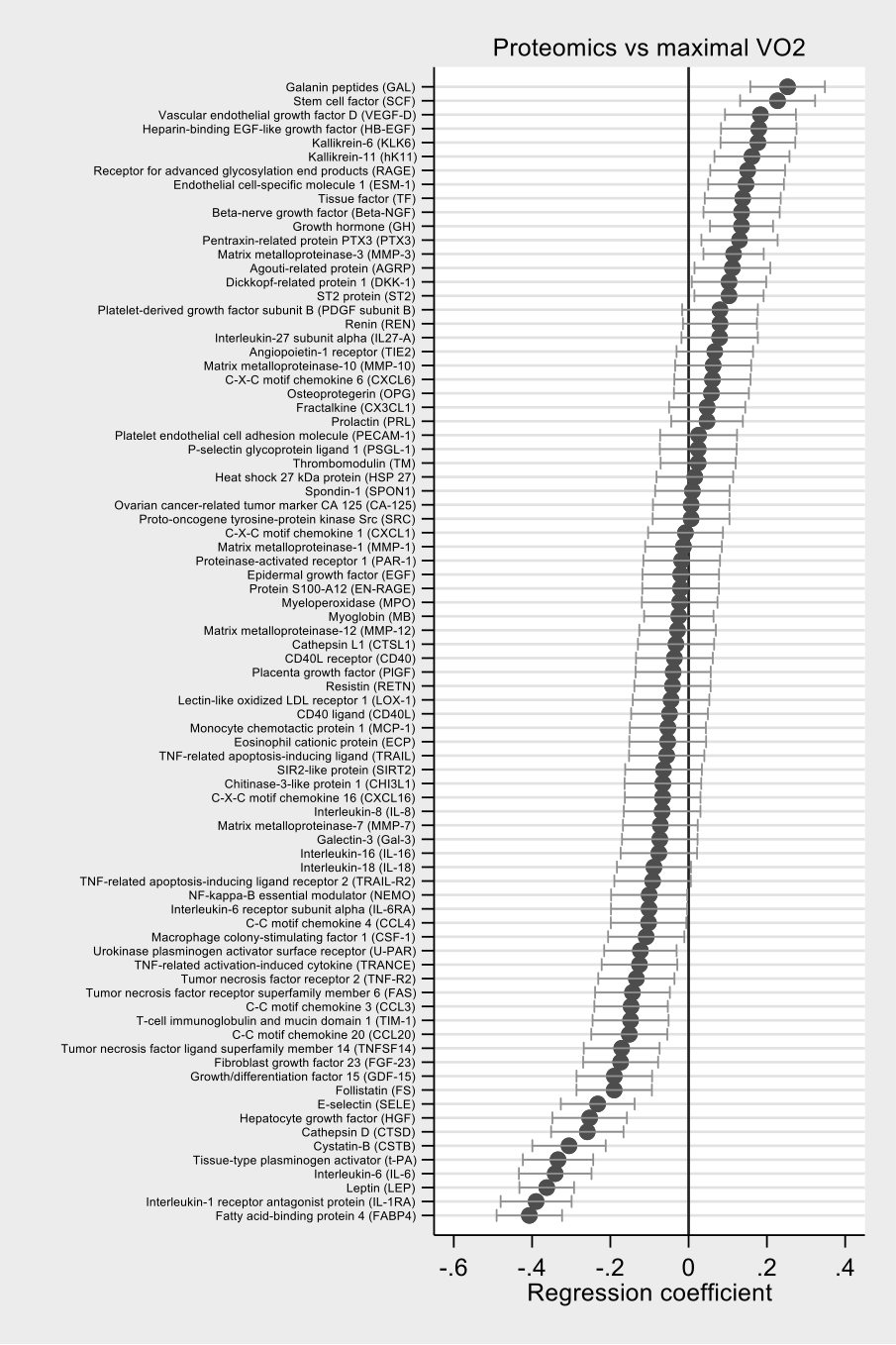

Supplement: Supplementary file 1 — Additional file 1. Supplementary figure 1. [file 40798_2021_343_MOESM1_ESM.docx]
