# Supplementary material for: Association between Cardiorespiratory Fitness and Circulating Proteins in 50-Year-Old Swedish Men and Women: a Cross-Sectional Study
Source: Sports Med Open. 2021 Jul 26;7:52. doi: 10.1186/s40798-021-00343-5 (PMC8313632; doi:10.1186/s40798-021-00343-5)
Supplement: Supplementary file 2 — Additional file 2. Supplementary figures 2–10. [file 40798_2021_343_MOESM2_ESM.docx]

**Sports Medicine Open**

**Association between cardiorespiratory fitness and circulating proteins in 50-year-old Swedish men and women.**

**Malin Enarsson**_a_**, Tobias Feldreich**_b_**, Liisa Byberg**_c_**, Christoph Nowak**_d_**, Lars Lind**_e_**, Johan Ärnlöv**_bd_

*_a_ Center for Clinical Research Dalarna, Uppsala University, Region Dalarna, Nissers väg 3, 79182, Falun, Sweden. malinanna.enarsson@regiondalarna.se*

***_b_*** *School of Health and Social Studies, Dalarna University, 79188, Falun Sweden*

*_c_ Department of Surgical Sciences, Orthopeadics, Uppsala University_,_ Dag Hammarskjölds väg 14 B 75185, Uppsala, Sweden.*

*_d_ Division of Family Medicine and Primary Care, Department of Neurobiology, Care Sciences and Society (NVS), Karolinska Institutet, Alfred Nobels Allé 23, SE 14183, Huddinge, Sweden. johan.arnlov@ki.se*

*_e_ Department of Medical Sciences, Uppsala University, Dag Hammarskölds väg 10B 75237, Uppsala, Sweden.*

**Corresponding author**

Johan Ärnlöv

Division of Family Medicine and Primary Care, Department of Neurobiology, Care Sciences and Society (NVS), Karolinska Institutet, Alfred Nobels Allé 23, SE 14183, Huddinge, Sweden.

Email: johan.arnlov@ki.se

**Supplementary Figure 2.** Spearman’s correlation coefficient for fatty acid-binding protein 4 (FABP4) and VO_2_peak.

**Supplementary Figure 3.** Spearman’s correlation coefficient for interleukin-6 (IL-6) and VO_2_peak.

**Supplementary Figure 4.** Spearman’s correlation coefficient for leptin and VO_2_peak.

**Supplementary Figure 5.** Spearman’s correlation coefficient for galanin and VO_2_peak.

**Supplementary Figure 6.** Spearman’s correlation coefficient for cystatin-B (CSTB) and VO_2_peak.

**Supplementary Figure 7.** Spearman’s correlation coefficient for interleukin-1 receptor antagonist protein (IL-1RA) and VO_2_peak.

**Supplementary Figure 8.** Spearman’s correlation coefficient for kallikrein-6 (KLK6) and VO_2_peak.

**Supplementary Figure 9.** Spearman’s correlation coefficient for heparin-bindning EGF-like growth factor (HB-EGF) and VO_2_peak.

**Supplementary Figure 10.** Spearman’s correlation coefficient for growth differentiation factor 15 (GDF15) and VO_2_peak.
