# Supplementary material for: Association between Cardiorespiratory Fitness and Circulating Proteins in 50-Year-Old Swedish Men and Women: a Cross-Sectional Study
Source: Sports Med Open. 2021 Jul 26;7:52. doi: 10.1186/s40798-021-00343-5 (PMC8313632; doi:10.1186/s40798-021-00343-5)
Supplement: Supplementary file 3 — Additional file 3. Supplementary table 1. [file 40798_2021_343_MOESM3_ESM.docx]

| **Sports Medicine Open**  **Association between Cardiorespiratory Fitness and Circulating Proteins in 50-year-old Swedish Men and Women: A Cross-sectional Study**  **Malin Enarsson**_a_**, Tobias Feldreich**_b_**, Liisa Byberg**_c_**, Christoph Nowak**_d_**, Lars Lind**_e_**, Johan Ärnlöv**_bd_  *_a_ Center for Clinical Research Dalarna, Uppsala University, Region Dalarna, Nissers väg 3, 79182, Falun, Sweden. malinanna.enarsson@regiondalarna.se*  ***_b_*** *School of Health and Social Studies, Dalarna University, 79188, Falun Sweden*  *_c_ Department of Surgical Sciences, Orthopeadics, Uppsala University_,_ Dag Hammarskjölds väg 14 B 75185, Uppsala, Sweden.*  *_d_ Division of Family Medicine and Primary Care, Department of Neurobiology, Care Sciences and Society (NVS), Karolinska Institutet, Alfred Nobels Allé 23, SE 14183, Huddinge, Sweden. johan.arnlov@ki.se*  *_e_ Department of Medical Sciences, Uppsala University, Dag Hammarskölds väg 10B 75237, Uppsala, Sweden.*  **Corresponding author**  Johan Ärnlöv  Division of Family Medicine and Primary Care, Department of Neurobiology, Care Sciences and Society (NVS), Karolinska Institutet, Alfred Nobels Allé 23, SE 14183, Huddinge, Sweden.  Email: johan.arnlov@ki.se  **Supplementary table 1.** Variance inflation factor (VIF) test for multicollinearity.  VIF model produces an R-squared value indicating percentage of variance in the individual independent factor that the set of independent factors explain, model does not include protein data. | | | |
| --- | --- | --- | --- |
|  |  |  |  |
| Variable | VIF | 1/VIF |  |
| Sex | 3.19 | 0.31 |  |
| Fat mass (DXA) | 2.58 | 0.39 |  |
| Waist-hip ratio | 1.66 | 0.60 |  |
| Systolic blood pressure | 1.13 | 0.88 |  |
| Low density lipoprotein (LDL) | 1.12 | 0.90 |  |
| Education | 1.05 | 0.96 |  |
| Smoking | 1.04 | 0.96 |  |
| Fasting glucose | 1.03 | 0.97 |  |
|  |  |  |  |
| Mean VIF | 1.54 |  |  |
|  |  |  |  |

VIF values <4 indicates a week correlation with the other independent variables, <1 no correlation.
