# Supplementary material for: Association between Cardiorespiratory Fitness and Circulating Proteins in 50-Year-Old Swedish Men and Women: a Cross-Sectional Study
Source: Sports Med Open. 2021 Jul 26;7:52. doi: 10.1186/s40798-021-00343-5 (PMC8313632; doi:10.1186/s40798-021-00343-5)
Supplement: Supplementary file 4 — Additional file 4. Supplementary table 2. [file 40798_2021_343_MOESM4_ESM.docx]

**Sports Medicine Open**

**Association between Cardiorespiratory Fitness and Circulating Proteins in 50-year-old Swedish Men and Women: A Cross-sectional Study**

**Malin Enarsson**_a_**, Tobias Feldreich**_b_**, Liisa Byberg**_c_**, Christoph Nowak**_d_**, Lars Lind**_e_**, Johan Ärnlöv**_bd_

*_a_ Center for Clinical Research Dalarna, Uppsala University, Region Dalarna, Nissers väg 3, 79182, Falun, Sweden. malinanna.enarsson@regiondalarna.se*

***_b_*** *School of Health and Social Studies, Dalarna University, 79188, Falun Sweden*

*_c_ Department of Surgical Sciences, Orthopeadics, Uppsala University_,_ Dag Hammarskjölds väg 14 B 75185, Uppsala, Sweden.*

*_d_ Division of Family Medicine and Primary Care, Department of Neurobiology, Care Sciences and Society (NVS), Karolinska Institutet, Alfred Nobels Allé 23, SE 14183, Huddinge, Sweden. johan.arnlov@ki.se*

*_e_ Department of Medical Sciences, Uppsala University, Dag Hammarskölds väg 10B 75237, Uppsala, Sweden.*

**Corresponding author**

Johan Ärnlöv

Division of Family Medicine and Primary Care, Department of Neurobiology, Care Sciences and Society (NVS), Karolinska Institutet, Alfred Nobels Allé 23, SE 14183, Huddinge, Sweden.

Email: johan.arnlov@ki.se

| **Supplementary table 2.** The associations between VO_2_peak normalized for lean mass, and the circulating proteins: sex- stratified analyses and multiplicative interaction analyses. | | | | | | | | | | | | |
| --- | --- | --- | --- | --- | --- | --- | --- | --- | --- | --- | --- | --- |
|  |  |  |  |  |  |  |  |  |  |  |  |  |
|  | **p for interaction** |  |  |  |  |  |  |  |  |  |  |  |
|  |  |  | **Women** | | | |  | **Men** | | | | |
|  |  |  |  |  |  |  |  |  |  |  |  |  |
|  |  |  | beta | 95%CI | 95%CI | p-value |  | beta | 95%CI | 95%CI | p-value |  |
| Protein |  |  |  | low | high |  |  |  | low | high |  |  |
|  |  |  |  |  |  |  |  |  |  |  |  |  |
| **Leptin** | 0.0005 |  | -0.27 | -0.36 | -0.18 | <0.0001 |  | -0.53 | -0.64 | -0.41 | <0.0001 |  |
| **Fibroblast growth factor 23 (FGF-23)** | 0.037 |  | -0.27 | -0.38 | -0.12 | 0.0003 |  | -0.039 | -0.18 | 0.10 | 0.59 |  |
| **Cathepsin D (CTSD)** | 0.018 |  | -0.18 | -0.28 | -0.07 | 0.0017 |  | -0.40 | -0.56 | -0.24 | <0.0001 |  |
| **Fatty acid-binding protein 4 (FABP4)** | 0.048 |  | -0.34 | -0.44 | -0.24 | <0.0001 |  | -0.52 | -0.66 | -0.38 | <0.0001 |  |
|  |  |  |  |  |  |  |  |  |  |  |  |  |
| Data are linear regression coefficients adjusted for age and sex | | | | |  |  |  |  |  |  |  |  |
